# Differential Gene Expression Analysis of SoCBL Family Calcineurin B-like Proteins: Potential Involvement in Sugarcane Cold Stress

**DOI:** 10.3390/genes13020246

**Published:** 2022-01-27

**Authors:** Bao-Qing Zhang, Xiu-Peng Song, Xiao-Qiu Zhang, Yu-Xin Huang, Yong-Jian Liang, Shan Zhou, Cui-Fang Yang, Li-Tao Yang, Xing Huang, Yang-Rui Li

**Affiliations:** 1 Key Laboratory of Sugarcane Biotechnology and Genetic Improvement (Guangxi), Ministry of Agriculture and Rural Affairs/Guangxi Key Laboratory of Sugarcane Genetic Improvement, Sugarcane Research Institute, Guangxi Academy of Agricultural Sciences, Nanning 530007, China; zbqsxau@126.com (B.-Q.Z.); xiupengsong@163.com (X.-P.S.); zhangxiaoqiuxhd@163.com (X.-Q.Z.); huangyuxin13@163.com (Y.-X.H.); shan1268225@126.com (S.Z.); yj993@163.com (C.-F.Y.); 2 College of Agriculture, Guangxi University, Nanning 530005, China; Yongjianliang_605@163.com (Y.-J.L.); liyr@gxu.edu.cn (L.-T.Y.)

**Keywords:** cold stress, calcineurin B-like protein, gene expression evolutionary analysis, qRT-PCR, sugarcane

## Abstract

Sugarcan e is a major crop for sugar and biofuel production and is cultivated in tropical and subtropical areas worldwide. Sugarcane growth is constrained because of winter’s low-temperature stress, and cold resistance is an important limitation in sugarcane growth enhancement. Therefore, in this study, we identified a gene involved in the low-temperature stress response of sugarcane. Calcineurin B-like (CBL) protein is a calcium signal receptor involved in the cold stress response. Five sugarcane CBL genes were cloned, sequenced, and named *SoCBL1*, *SoCBL3*, *SoCBL5*, *SoCBL6*, and *SoCBL9*. The protein sequences of these genes were analyzed. The calculated molecular weight of these proteins was 24.5, 25.9, 25.2, 25.6, and 26.3 kD, respectively. Subcellular localization analysis revealed that SoCBL1, SoCBL3, SoCBL6, and SoCBL9 were situated in the cytoplasm, while SoCBL5 was present in mitochondria. Secondary structure analysis showed that these five CBL proteins had similar secondary structures. Conserved domain analysis displayed that each sugarcane CBL protein contained three conserved EF domains. According to the self-expanding values of the phylogenetic tree, the CBL gene family was divided into four groups. The *CBL1* and *CBL9* genes were classified into one group, illustrating that these two genes might possess a similar function. The expression analysis of the *SoCBL* gene under low temperatures showed that *SoCBL3* and *SoCBL5* were affected significantly, while *SoCBL1* and *SoCBL9* were less affected. These results demonstrate that the *CBL* genes in sugarcane have similar characteristics and present differences in genetic diversity and gene expression response to low temperatures. Therefore, these genes might be novel candidates for fighting cold stress in sugarcane.

## 1. Introduction

Plants are constantly confronted with various abiotic stresses, such as drought, extreme temperatures, and salinity, during their growth. In order to adapt to environmental changes and self-protection, a complex signal transmission network system has been formed in the process of evolution to perceive and respond to environmental stress. Many stress-responsive genes receive these stress signals and notice their expression at specific times, prompting tissues to undergo physiological changes to adapt to the environment [1]. Among these changes, calcium signal transduction in plants is an essential signaling pathway for stress transmission by enhancing the calcium concentration in vivo in plants [2,3]. Thus far, three calcium signal sensors, including calmodulin (CaM), calcium-dependent protein kinase (CDPK), and calcineurin B-like protein (CBL), have been found in plant cells [4,5,6,7]. Signal transduction occurs between calcium signals and their interacting proteins to regulate the expression of downstream response genes, indicating that calcium signaling is a key regulatory pathway in the plant response to stress.

Calcineurin B-like (CBL) protein is a type of calcium signal receptor specific to plants. The core region of the plant CBL protein is relatively conserved, including four EF-hand motifs with the characteristic Ca^2+^ affinity and Ca^2+^ binding [8], but variations of EF-hand domain sequences occur in different CBLs [9]. Each CBL protein interacts with a downstream kinase harboring 26 serine/threonine, forming a CBL-interacting protein kinase (CIPKs) [10]. Subsequently, the activated CIPKs rapidly phosphorylate the downstream signaling molecules to build an entire signaling network to transmit calcium signals [11]. CBL1 plays an essential role in various abiotic stress pathways, while CBL4/SOS3 is involved in mediating ion tolerance [11,12].

At present, several studies have confirmed that CBL is involved in the external environment’s effect on plants. Indeed, in Arabidopsis, *AtCBL1* is an upstream regulator of stress response genes [13]. *AtCBL9* overexpression produced enhanced tolerance to drought [14]. *AtCBL9* mutant plants became hypersensitive to ABA at the early developmental stages, whereas it attenuated the response to drought and salinity. On the contrary, *AtCBL9* overexpression increased stress tolerance, indicating that CBL is involved in the plant response and the regulation of stress signals [15]. Rice *OsCBL1* and *OsCBL3* respond to ABA, NaCl, and low-temperature stress [16]. The pea *PsCBL* gene was involved in responsiveness to salt/alkali, drought, and low-temperature stress [17]. The expression of *SiCBL1*, *SiCBL3*, and *SiCBL5* in millet was increased under drought and salt stress [18]. To date, there have been few reports on *CBL* genes in sugarcane [19,20,21,22]. Farani et al. [19] characterized the protein–protein interactions of ScCIPK8 with six CBL proteins (ScCBL1, ScCBL2, ScCBL3, ScCBL6, ScCBL9, and ScCBL10). Ling et al. [20] found that SsCBL1 and SsCBL6 play important regulatory roles in response to a variety of stresses (low potassium, drought, and salt). According to datasets of the transcription sequencing of sugarcane genes under abiotic stress, Ling et al. [21] cloned a calcineurin B-like gene named *SsCBL4* (GenBank No. KY674 987.1) from sugarcane root. Su et al. [22] obtained three *CBL* genes (GenBank accession nos. KX013374, KX013375, and KX013376) from sugarcane variety ROC22. These *ScCBL* genes were constitutively expressed in the sugarcane bud, stem pith, leaf, meristem, and stem skin, and they showed different expression patterns in response to stimulation with phytohormones and various abiotic stresses.

In order to understand the relationship between *CBL* genes and low-temperature stress in sugarcane, the mRNAs of five sugarcane *CBL* genes were cloned and sequenced. Bioinformatics and genetic diversity analyses of the translated protein sequences were performed. Real-time PCR was used to study the expression pattern of these genes in response to low temperatures. A low temperature has a profound effect on increasing the expression of *CBL* genes. The results might provide an insight into the roles of *CBL* genes in low-temperature resistance in sugarcane.

## 2. Materials and Methods

### 2.1. Plant Material and Growth Conditions

The cold-resistant sugarcane cultivar GT28 and the cold-susceptible cultivar YL6 used in this experiment were supplied by the Sugarcane Research Institute, Guangxi University.

In a greenhouse, the single-bud seedcane sets were planted on a mixed substrate of soil and sand. When the plants reached three leaves, those with consistent growth were selected and transplanted for soil culture in pots in a randomized design. The pots were 300 mm in diameter and 350 mm in height and filled with 17.5 kg culture medium (soil–organic fertilizer–sand = 70–20–10 in weight). Each pot had 2–3 plants. In order to enhance permeability, holes were punched in the bottom of the pots. The plants were managed routinely. After 10 months of normal growth, when the plants were at the sugar-accumulating stage, low-temperature treatment was applied. For treatment, the plants were divided into two groups. The first group was grown normally, as the control, with a temperature of 25 °C, a light intensity of 250–300 µmol/m^2^·s, a 12 h photoperiod, and relative humidity of 60–70%. The second group was treated with a low-temperature set at 0–4 °C, light intensity at 250–300 µmol/m^2^·s, 12 h photoperiod, and relative humidity at 60–70%. After 2, 4, 6, and 8 days of low-temperature stress, the leaf and stem tip samples were collected at 8:00 am, quick-frozen in liquid nitrogen, and stored at −80 °C.

### 2.2. cDNA First-Strand Synthesis

The shoots were frozen in liquid nitrogen and ground into powder. Total RNA was extracted using Trizol (Invitrogen, Gaithersburg, MD, USA) as described by the manufacturer. RNA concentration and integrity were determined using NanoDrop (Thermo Fisher, Saint Luis, MO, USA) and agarose gel electrophoresis, respectively. The first strand of cDNA for gene cloning was synthesized using the M-MLV cDNA first-strand synthesis kit (Takara, DaLian, China) according to the protocol provided by the manufacturer. Then, the cDNA was diluted to the concentration of 100 μg/μL for further gene cloning PCR. Finally, cDNA synthesis for qRT-PCR was carried out using the PrimeScript RT Reagent Kit with gDNA Eraser (Takara, DaLian, China) according to the provided protocol.

### 2.3. PCR Primer Design and Amplification Conditions

In order to find the conserved sequence for primer design, highly homologous nucleic acid sequences of *CBL* genes were selected for the alignment analysis using DAMAN software. The forward and reverse primers of the target gene were designed for cloning (Table 1). The PCR reaction mixture (25 μL) was as follows: mixture cDNA template 1 μL, 10 × buffer 2 μL, forward primer (10 mM) 1 μL, reverse primer (10 mM) 1 μL, Taq DNA polymerase (200 U/μL) 0.16 μL, dNTPs (10 mM) 0.4 μL, and ddH_2_O 19.44 μL. PCR reaction was performed for 35 cycles of denaturation, annealing, and elongation at 95 °C for 40 s, 55 °C for 50 s, and 72 °C for 2 min. After the reaction, 4 μL of PCR products was validated by electrophoresis on 1.0% agarose gel. The PCR products were gel-purified and cloned into a pMD18-T vector as described by the manufacturer (Takara, DaLian, China). The clone was sequenced using the ABI 3730XL genetic analyzer (Thermo Fisher, Saint Luis, MO, USA).

### 2.4. Sequence Analysis and Prediction

The nucleotide sequences of the *CBL1*, *CBL3*, *CBL5*, *CBL6*, and *CBL9* genes in sugarcane were analyzed via various analytical software programs and tools. BioXM 2.6 was used to predict the gene-encoded amino acid sequence. NCBI online analysis software was used for gene homology analysis compared to other species. The protein molecular weight and isoelectric point were analyzed using online software (http://isoelectric.ovh.org/, accessed on 3 December 2021). Protein subcellular localization was analyzed using the WoLF PSORT online software. SOSUI signal software was used for prediction of the signal peptide. Protein secondary structure was predicted by the SOPMA software. The protein motif was analyzed using Motif Scan online software. MEGA 4.0 software was used to construct a phylogenetic tree for each gene-encoded amino acid sequence compared to other species.

### 2.5. Real-Time Quantitative PCR for Gene Expression Analysis

Specific primers for real-time PCR were designed based on the full-length cDNA sequences of *CBL1*, *CBL3*, *CBL5*, *CBL6*, and *CBL9* genes (Table 2). The sugarcane glyceraldehyde 3-phosphate dehydrogenase (GAPDH) gene (EF189713) was used as the reference gene. The real-time PCR reaction was performed on a Light Cycler 480II (Roche, Switzerland) real-time PCR instrument, and the data were analyzed using qBASE plus software. The qRT-RCR reaction mixture (20 μL) included SYBR^®^ PremixExTaqTMII (2×) 10 μL, forward primer (10 μM) 0.8 μL, reverse primer (10 μM) 0.8 μL, cDNA template of each sample (50 ng/μL) 2.0 μL, ddH_2_O 6.4 μL. The qRT-RCR reaction procedure was as follows: pre-incubation 1 cycle at 95 °C for 30 s, amplification 45 cycles at 95 °C for 5 s and 60 °C for 20 s, melting curve 1 cycle at 95 °C for 1 s and 65 °C for 15 s, 95 °C continuous, and cooling at 40 °C for 30 s. The relative expression was calculated by the 2^−ΔΔCt^ method with three biological replicates.

### 2.6. Statistical Analysis

The data were analyzed using SPSS 15.0 software, and the significant difference between treatments was tested with Duncan’s new multiple range method (*P* ≤ 0.05).

## 3. Results

### 3.1. Total RNA and cDNA Quality of Sugarcane Leaves

**Figure 1:**
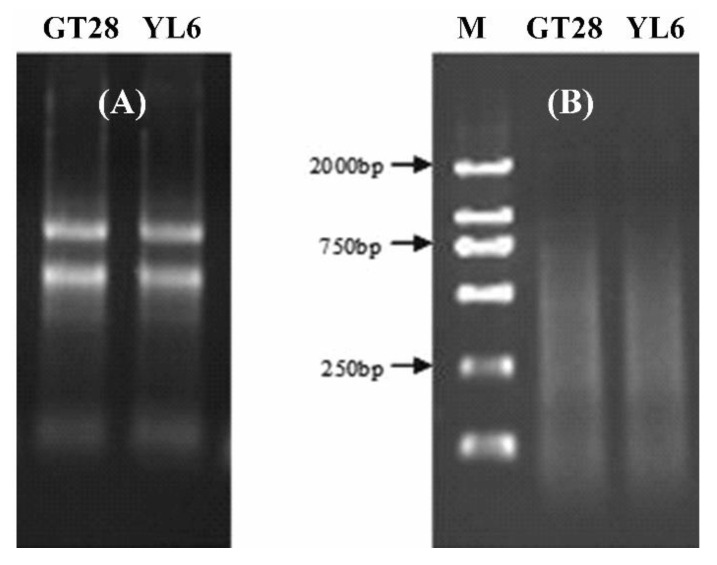
The quality of total RNA and cDNA from leaves of sugarcane cultivars GT28 and YL6. (**A**) The total RNA was detected by 1.0% gel agarose gel electrophoresis. (**B**) The size of the cDNA products was between 100 and 2000 bp. M, molecular weight marker.

The total RNA in sugarcane leaves was extracted using Trizol, and the concentration and purity of the total RNA were detected by 1.0% agarose gel electrophoresis and UV spectrophotometry (Figure 1A). The results showed that the OD260/280 value was between 1.85 and 1.95, and the OD260/230 was between 2.0 and 2.2, indicating that the purity and quality of the extracted RNA were high. The RNA was used to synthesize cDNA using a reverse transcription kit, and the size of the cDNA products was between 100 and 2000 bp, illustrating that the reverse transcription quality was good (Figure 1B). Therefore, the total RNA and cDNA obtained in the experiment were good enough for the subsequent experiments.

### 3.2. Cloning and Sequencing of Sugarcane SoCBL Genes

**Figure 2:**
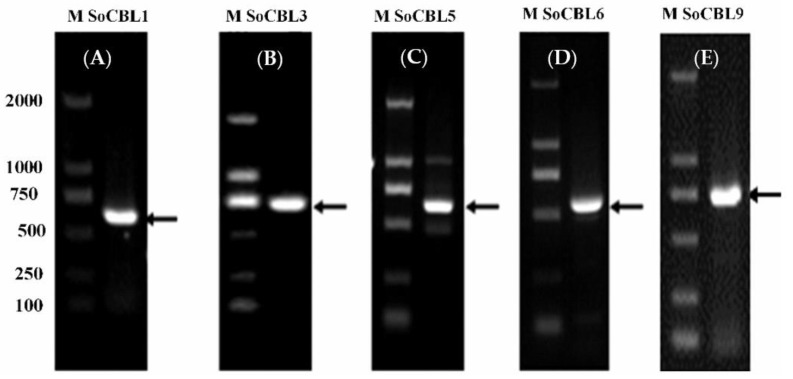
PCR product analysis of the five *SoCBL* genes (**A**): *SoCBL1*; (**B**): *SoCBL3*; (**C**): *SoCBL5*; (**D**): *SoCBL6*; (**E**): *SoCBL9* in sugarcane. M, molecular weight marker.

According to the literature, the *CBL* genes are related to cold tolerance [1,13,17,23,24]. Therefore, from the NCBI database, we referred to the *CBL* gene nucleic acid sequences of sorghum and maize to design the upstream and downstream degenerate primers for *CBL1*, *CBL3*, *CBL5*, *CBL6*, and *CBL9*, respectively. Then, using cDNA as the template and the upstream and downstream primers of the above five genes, the fragments of 642, 678, 657, 672, and 693 bp were amplified, respectively (Figure 2), and they were recovered, cloned, and sequenced. After comparison, it was found that the cDNA lengths were the same as those of *CBL1*, *CBL3*, *CBL5*, *CBL6*, and *CBL9*. Therefore, they were named *SoCBL1*, *SoCBL3*, *SoCBL5*, *SoCBL6*, and *SoCBL9*, respectively, and the NCBI gene registration numbers were KC800815, KC800816, KC800817, KC800818, and KC800819, respectively.

### 3.3. Bioinformatics Analysis of Sugarcane SoCBL

**Figure 3:**
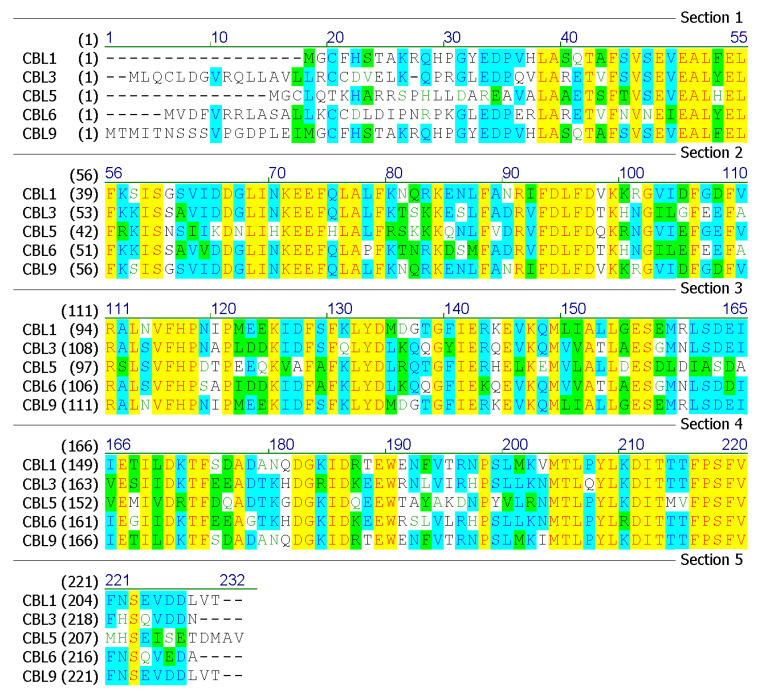
Predicted amino acid sequences of the SoCBL proteins in sugarcane.

Bioinformatics analyses of the amino acid sequences of sugarcane SoCBL proteins (SoCBL1, SoCBL3, SoCBL5, SoCBL6, and SoCBL9) were performed (Figure 3).

The protein molecular weights of the five proteins were 24.5, 25.9, 25.2, 25.6, and 26.3 kD, respectively, and their corresponding isoelectric points were 4.6, 4.6, 5.0, 4.7, and 4.5, respectively (Table 3).

In addition, the subcellular localization analysis of the SoCBL proteins using online software revealed that SoCBL1, SoCBL3, SoCBL6, and SoCBL9 were mainly located in the cytoplasm, while SoCBL5 was mainly located in mitochondria. No signal peptide was detected in SoCBL1, SoCBL5, SoCBL6, and SoCBL9. The sequence of SoCBL3 contained a signal peptide (MLQCLDGVRQLLAVLLRCCD) located at the C-terminus. No trans-membrane motifs were aligned in any of the five proteins using TMpred. In order to further understand the structure and function of the SoCBL proteins, prediction of the protein secondary structure was performed using SOPMA. The analysis result showed that, even with different sequences, the amount and ratio of each putative secondary structure were relatively similar (Table 4).

In order to identify the regulation of protein expression, we performed some post-translation modification site analyses on the SoCBL proteins (Table 5). The numbers of putative casein kinase II phosphorylation sites in SoCBL1, SoCBL3, SoCBL5, SoCBL6, and SoCBL9 were six, seven, five, four, and four, respectively. There was one putative N-myristoylation site in each SoCBL protein. SoCBL1 and SoCBL5 had similar putative sites in N-myristoylation, while SoCBL3 shared alike putative N-myristoylation sites and cAMP- and cGMP-dependent protein kinase phosphorylation putative sites with SoCBL6. In the EF calcium-binding domain and EF domain analyses, all SoCBL proteins contained the same number and similar location of these two putative domains.

### 3.4. Phylogenetic Analysis of Sugarcane SoCBL

**Figure 4:**
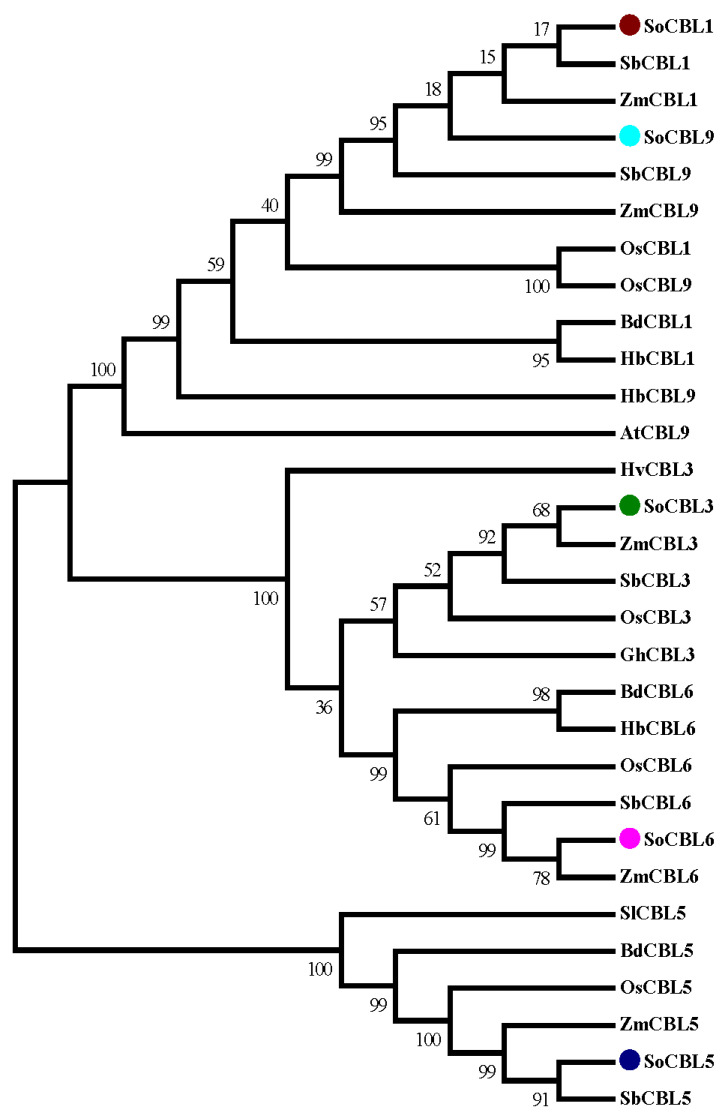
Amino acid sequence homology analysis of the SoCBL proteins from sugarcane compared with the CBL proteins from other plant species. SoCBL1, *Saccharum officenarum*; SbCBL1, *Sorghum bicolor* XP_002467472.1; ZmCBL1, *Zea mays* NP_001130480.1; OsCBL1 *Oryza sativa* Q7XC27.2; BdCBL1, *Brachypodium distachyon* XP_003574350.1; HbCBL1, *Hordeum brevisubulatum* AER42616.1; SoCBL3, *Saccharum officenarum*; SbCBL3, *Sorghum bicolor* XP_002442450.1; ZmCBL3, *Zea mays* NP_001131294.1; OsCBL3, *Oryza sativa* EEC69608.1; HvCBL3, *Hordeum vulgare* BAJ86631.1; GhCBL3, *Gossypium hirsutum* ABW06390.1; SoCBL5, *Saccharum officenarum*; SbCBL5, *Sorghum bicolor* ACQ83549.1; ZmCBL5, *Zea mays* DAA58834.1; OsCBL5, *Oryza sativa* BAD53426.1; BdCBL5, *Brachypodium distachyon* XP_003568025.1; SlCBL5, *Solanum lycopersicum* NP_001234705.1; SoCBL6, *Saccharum officenarum*; BdCBL6, *Brachypodium distachyon* XP_003563139.1; OsCBL6, *Oryza sativa* NP_001066223.1; ZmCBL6, *Zea mays* NP_001151206.1; SbCBL6, *Sorghum bicolor* XP_002442860.1; HbCBL6, *Hordeum brevisubulatum* AER42617.1; SoCBL9, *Saccharum officenarum*; ZmCBL9, *Zea mays* NP_001151319.1; OsCBL9, *Oryza sativa* Q7XC27.2; HbCBL9, *Hordeum brevisubulatum* AFD23460.1; AtCBL9, *Arabidopsis thaliana* NP_199521.1; SbCBL9, *Sorghum bicolor* XP_002467472.1.

In order to investigate the evolutionary relationship of the sugarcane CBL protein family, phylogenetic analysis of the five sugarcane CBL protein sequences was performed. Twenty-four CBL proteins from different plants were retrieved from the GenBank database. All the protein sequences were aligned and trimmed. The resulting multiple sequence alignment (MSA) was used for phylogenetic analysis. The analysis showed that the CBL protein family was divided into four main clades (Figure 4).

All the sequences of CBL1 and CBL9 were clustered into one clade, indicating that these two members have high sequence identity. Clade II contained all the CBL6 proteins of all the species with strong support (bootstrap percentages, BP = 100). Clade III comprised all the CBL3 proteins from all the species (BP = 100). All the CBL5 proteins were supported in clade IV but with lower bootstrap percentages (BP = 50). Clades II and IV were the nodes with strong support (BP = 100), demonstrating that CBL3 and CBL6 are genetically similar. Each of the same types of genes was clustered into different clades, indicating that different clades of the CBL protein family may present different functions. Within the main clade, the sugarcane (*Saccharum officinarum*, So) proteins were always clustered with the proteins from sorghum (*Sorghum bicolor*, Sb) and maize (*Zea mays*, Zm) (Figure 4). The CBL proteins from rice (*Oryza sativa*, Os) were correlated more to sugarcane than those from brome (*Brachypodium distachyon*, Bd) and *Hordeum brevisubulatum* in sequence diversity.

### 3.5. Change in Expression of SoCBL Genes in Response to Low-Temperature Stress

In order to understand the relationship of low-temperature stress with *SoCBL* gene expression, the relative change in mRNA accumulation upon exposure to 4 °C for 2, 4, 6, and 8 days was measured.

**Figure 5:**
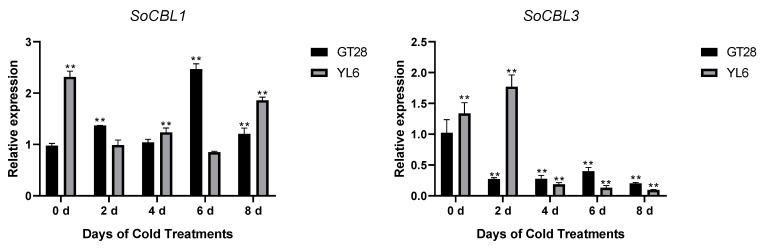

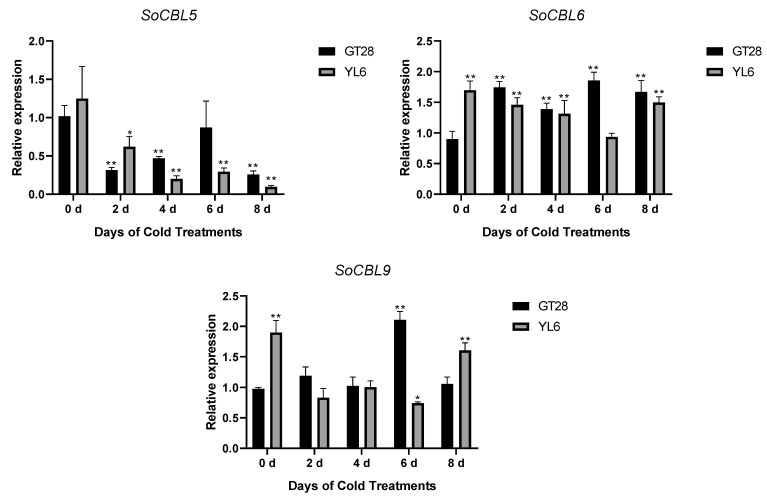
Expression analysis of the *SoCBL* genes in the leaves of sugarcane cultivars GT28 (cold-resistant) and YL6 (cold-susceptible) under low-temperature stress. All the tested results were obtained from 3 biological replicates and statistical analysis was applied via Student’s *t*-tests in GraphPad Prism 8.0 software; *p* < 0.05 for significant difference (*) and *p* < 0.01 for highly significant difference (**).

The results showed that the expression levels and trends of *SoCBL1* and *SoCBL9* genes were consistent in the cold-resistant cultivar GT28 and the cold-susceptible cultivar YL6 under low-temperature stress. The relative expression level of *SoCBL1*, *SoCBL5*, *SoCBL6*, and *SoCBL9* increased, while *SoCBL3* showed almost no change from 2 to 6 days of low-temperature stress in the cold-resistant cultivar GT28. For the cold-susceptible cultivar YL6, however, the highest expression of these genes was found in the control, except for *SoCBL3*. After 2 days of low-temperature stress, the expression of *SoCBL3* and *SoCBL5* declined rapidly, and then showed a steady decline, and reached the lowest level at 8 days in cold-susceptible cultivar YL6 (Figure 5).

**Figure 6:**
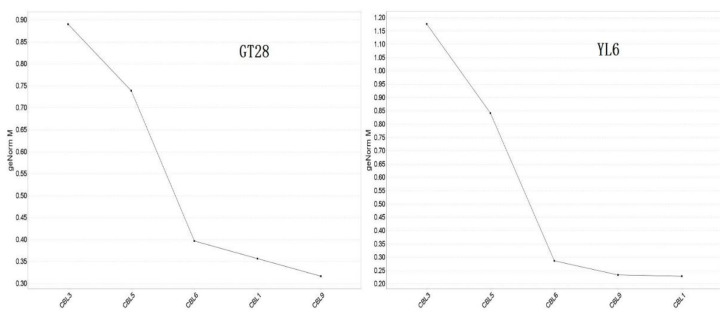
Average expression stability of sugarcane *SoCBL* genes under low-temperature stress.

In addition, the relative expression stability of these five *SoCBL* genes under low-temperature stress was analyzed using qBASE plus software, and the results (Figure 6) indicated that the higher the expression value, the more affected the plant was under low-temperature stress.

All five genes were affected by the low-temperature stress in both varieties, with *SoCBL3* being affected the most, followed by *SoCBL5*, while *SoCBL1* and *SoCBL9* were less affected, which indicates that the role of the *CBL* genes was consistent regardless of varieties under low-temperature stress.

## 4. Discussion

Cold is a detrimental abiotic stress to the growth of sugarcane, decreasing the crop yield during the cold season and affecting the income from crop production [25,26]. The cold resistance of sugarcane is already known to be associated with morphological and physiological characteristics such as leaf thickness, vesicular cell area, thick-walled vascular bundle tissue thickness, and electrolyte extravasation rates [27]. In addition, cold stress leads to increased peroxidation of the leaf membrane, and increased soluble sugar, proline, and MDA content, but decreased chlorophyll content in sugarcane [28,29]. At a molecular level, studies revealed that cold stress influenced the expression of several genes in sugarcane, including those identified by bioinformatic analyses to be involved in calcium metabolism [30,31]. Similar results were reported in other plant species [32,33,34].

Calcium is a critical messenger in many adaptation and developmental processes, and is detected and transmitted by a variety of calcium sensor molecules to elicit different responses in plants. The calcineurin B-like protein (CBL) family serves as a unique group of calcium sensors by sensing calcium signals with the help of a family of protein kinases (CIPKs). The five *SoCBL* genes (*SoCBL1*, *SoCBL3*, *SoCBL5*, *SoCBL6*, and *SoCBL9*) were cloned in the present study. The amino sequences and functional domains were analyzed. The structures of the five CBL proteins were very conserved and identical (Table 3). All five proteins contained three EF-hand domains, whereas it was reported that the CBL protein typically contains four highly conserved EF-hand domains [35]. These two domains might be involved because the EF-hand domain binds Ca^2+^ via the loop structure to cause protein conformation changes, thereby activating the protein function and then regulating plant responses to various stresses with the help of multiple signal transduction systems [9]. In addition, it was found that the sugarcane SoCBL proteins contain various putative phosphorylation sites, such as casein kinase II and protein kinase C, especially in SoCBL3, SoCBL5, and SoCBL6, which also contain putative cAMP- and cGMP-dependent protein kinase phosphorylation sites. When plants undergo environmental changes, the Ca^2+^ receptor itself does not perform an enzymatic activity, but its activity is activated when binding Ca^2+^ combined with the Ca^2+^ released by the plant itself to activate the Ca^2+^-regulated target enzyme activity, subsequently by phosphorylation and a series of intermolecular interactions of kinases, regulating the expression changes of specific genes to enhance stress resistance [36]. It was shown that this EF-hand domain of CBL plays a role in salt tolerance [37,38]. However, further investigation is needed to investigate whether it has the same effect under low-temperature stress.

Numerous studies have shown that CBL plays an important regulatory role in plants under many environmental stresses, such as high salt and low temperature [39], but few studies have examined the *CBL* genes in sugarcane [19,20,22,40]. Five *CBL* genes were cloned (GenBank accession #KC800815, KC800816, KC800817, KC800818, and KC800819) from the sugarcane cultivar GT28 by Zhang in 2013 [40]. The *CBL* members in sugarcane appear to be involved in the response to numerous chemical stresses, such as salicylic acid, methyl jasmonate, hydrogen peroxide, polyethylene glycol, salt, and copper chloride [22]. The overexpression of *CBLs* in *Nicotiana benthamiana* also increased the tobacco resistance to the *Ralstonia solanacearum* pathogen [22]. Ling et al. [20] also showed that *ScCBL1* and *ScCBL6* were involved in low-potassium, drought, and salt stresses. In this research, the expression analysis of five sugarcane *CBL* genes under low-temperature stress was carried out, and it was found that the expression of *SoCBL1*, *SoCBL5*, *SoCBL6*, and *SoCBL9* increased under low-temperature stress, indicating that these genes play an important role in plant signal transduction under low-temperature stress. Because the basic function of *CBL* is to sense the changes in calcium concentration, the expression of sugarcane *CBL* genes probably increased, triggered by the calcium concentration, suggesting that these genes can respond to the changes in calcium concentration [41]. Therefore, it is speculated that the *CBL* genes in sugarcane might participate in the response to low-temperature stress. Nevertheless, the expression of *SoCBL3* declined under low-temperature stress, suggesting that the regulation mechanism of the *CBL* family genes is different under different types of temperature stress.

## 5. Conclusions

A CBL-CIPK signaling pathway is a complex system. In order to make clear how the sugarcane *CBL* genes interact with specific proteins to activate the downstream genes under low-temperature stress, a comprehensive analysis of genes was needed concerning the function of sugarcane CBL and CIPK. The five sugarcane *CBL* genes cloned in this study showed a certain response to low-temperature stress, but, due to different gene functions, the defense pathways involved in the response to low-temperature stress might be different, which would provide an important molecular basis for strengthening sugarcane against low-temperature stress and other abiotic stresses, and could be referred to by subsequent research.

## Figures and Tables

**Table 1:** PCR primers used for gene cloning analysis.

Primer	Sequence (5′-3′)	Size of Fragment (bp)
CBL1-F	ATGGGGTGCTTCCATTCCACGGCGA	642
CBL1-R	TCACGTGACGAGATCGTC(C/G) ACTTC	
CBL3-F	ATG(T/G)TGCAGTGCCTGGA(T/C) GG	678
CBL3-R	TCAG(T/G)TATCATCGAC(T/C)TGAGA	
CBL5-F	ATGGGCTGTCTGCAAACAAAGCACG	657
CBL5-R	TTAGACAGCCATGTCTGTTTC	
CBL6-F	ATGGTGGACTT(T/C) GTTCGACGGCT	672
CBL6-R	TCACGCATCCTCTACTTG(A/C) GAGTTG	
CBL9-F	ATGGG(A/G) TGCTTCCATTCCACGGC	693
CBL9-R	TCACGTGACGAGATC(A/G) TC(G/C) ACTTC	

**Table 2:** Primers used for real-time PCR analysis.

Primer	Sequence (5′-3′)	Size of Fragment (bp)
GAPDH-F	AAGGGTGGTGCCAAGAAGG	145
GAPDH-R	CAAGGGGAGCAAGGCAGTT	
CBL1-F	TATGGATGGCACAGGGTTTATT	131
CBL1-R	CAGCGTCCGAAAATGTCTTATC	
CBL3-F	AGCAAGAAGGAGAGCCTGTTC	123
CBL3-R	AAGGGGAGCATTAGGATGAAAT	
CBL5-F	ACACAAAAGGTGATGGGAAGAT	98
CBL5-R	CTTGAGGTAGGGAAGGGTCATA	
CBL6-F	GTGGTTGATGATGGCTTGATTA	172
CBL6-R	CACTTGGATGGAACACAGAAAG	
CBL9-F	TATGGATGGCACAGGGTTTATT	131
CBL9-R	CAGCGTCCGAAAATGTCTTATC	

**Table 3:** Identification of the five SoCBL proteins in sugarcane.

Protein	Accession Number	MW	pI
SoCBL1	AGO81718.1	24.5 kD	4.6
SoCBL3	AGO81719.1	25.9 kD	4.6
SoCBL5	AGO81720.1	25.2 kD	5.0
SoCBL6	AGO81721.1	25.6 kD	4.7
SoCBL9	AGO81722.1	26.3 kD	4.5

**Table 4:** Putative functional domain analysis of amino acid sequences encoded by five SoCBL proteins in sugarcane.

Protein	Casein Kinase II Phosphorylation Site	N-Myristoylation Site	Phosphorylation Site	N-Glycosylation Site	CAMP- and cGMP-Dependent Protein Kinase Phosphorylation Site	EF Calcium-Binding Domain
SoCBL1	28~31, 45~48, 151~154, 156~159, 171~174, 206~209	2~7	7~9, 112~114			161~173, 67~102, 104~139,
SoCBL3	42~45, 79~82, 150~153, 165~168, 170~173, 176~179, 220~223	155~160		157~160, 200~203	54~57, 80~83	79~114, 116~151, 160~195
SoCBL5	31~34, 106~109, 143~146, 165~168, 212~215	2~7	67~69	189~192	43~46	70~105, 107~142, 151~186
SoCBL6	148~151, 168~171, 174~177, 218~221	153~158	76~78	155~158, 198~201	52~55, 78~81	79~114, 116~151, 160~195
SoCBL9	45~48, 62~65, 168~171, 173~176, 188~191, 223~226	19~24	24~26, 129~131	6~9		84~119, 121~156, 165~200

**Table 5:** Putative secondary structure of the five SoCBL proteins in sugarcane.

Protein	Number of α-Helices	Number of Extended Strands	Number of β-Turns	Number of Random Coils
SoCBL1	106 (49.8%)	16 (7.5%)	14 (6.6%)	77 (36.2%)
SoCBL3	126 (56.0%)	13 (5.8%)	18 (8.9%)	68 (30.2%)
SoCBL5	110 (50.5%)	23 (10.6%)	14 (7.2%)	71 (32.7%)
SoCBL6	121 (54.3%)	13 (5.8%)	16 (7.2%)	73 (32.7%)
SoCBL9	118 (51.3%)	19 (8.3%)	16 (7.0%)	77 (33.5%)
